# Supplementary material for: Learning curve and surgical outcome of robotic assisted colorectal surgery with ERAS program
Source: Sci Rep. 2022 Nov 29;12:20566. doi: 10.1038/s41598-022-24665-w (PMC9709162; doi:10.1038/s41598-022-24665-w)
Supplement: Supplementary file 1 — Supplementary Information 1. [file 41598_2022_24665_MOESM1_ESM.zip › LSC Raw data-Ñ[▒K/083/P_ñw╝╨░O▒Kñσ.pdf]

臺中榮民總醫院 臺中市臺灣大道四段[illegible] 病理部[illegible]活體組織[illegible] 臨床醫學實驗及病理診斷中心 檢驗(查)報告

[redacted]

Pathologic diagnosis:
Sigmoid colon, colectomy with rectopexy --- Focal mucosal erosion, congestion, muscular and nerve bundles hypertrophy, consistent with prolapse.

Gross description:
The specimen consists of a segment of unopened rectum, 13 cm in length and 3.4/7 cm in bilatera cut end circumferences. On cut, no obvious tumor is found grossly. Representative part for section: A-B) cut ends C-H) random colon tissue.
#T-59472_2 #M-31050_2 0 1400 000000

已電子簽章

敬告：本單僅供醫師參考使用，不具任何申請效力！！
請病患妥為保管，若不慎遺失需自負個人資料外洩責任！
